# Repairing Fractured Teeth

**Published:** 1904-06

**Authors:** C. Edmund Kells

**Affiliations:** New Orleans, La.


### REPAIRING FRACTURED TEETH.

By D8. C. Edmund Kells, Jr., New Orleans, La.

In "The Principles and Practice of Crowning Teeth," recently published by Dr. H. J. Goslee, I note that his treatment of these cases is so unlike the method devised and used successfully by myself that I am led to offer it for the consideration of those who are interested in the subject.

My early cases happened to be men and the teeth (molars) apparently too good to be cut off and crowned, and in these cases narrow gold bands were fitted to the crowns and cemented in place, thus preserving them for many years in some instances. This method, however, I do not believe was original with me.

But several years ago, a gentleman presented with a bicuspid split in two, and the crown was comparatively good. A gold band around it as just described would have been too conspicuous; a gold crown was not considered, as the making of crowns of that character had been abandoned some years previously, and a porcelain crown was practically a physical impossibility. I was in a quandary.

It then occurred to me to bolt the two pieces together, as any mechanic would do were he confronted with a like proposition in his own line, and I proceeded as follows:

A piece of iridio-platinum wire of suitable length and diameter was threaded with the tap furnished with the Bryant bridge repair set, and upon one end was soldered one of the Bryant cone-shaped nuts.

The two parts having been previously brought together with brass ligature wire, as shown by Dr. Goslee, a hole was drilled through the tooth from the buccal aspect, and of such a size as would allow the threaded wire to just slip through. The openings upon both buccal and lingual surfaces were then countersunk, so as to allow of the nuts being sunk into the tooth.

The bolt was now passed through the tooth, a second nut (also reduced to proper size) was put on the other end and the two parts brought firmly together, the tooth having been previously dried and the bolt and nuts coated with chloro-percha.

When the nuts were polished down to conform to the surface of the teeth, they appeared to be small fillings, and the buccal one, the only one visible, was quite inconspicuous.

This tooth was seen some eighteen months after this work was done and then appeared as solid as before being fractured. Since then a lady presented with the first upper molar in same condition and it was treated in same manner, and at last accounts the bolts had proven a success.

It is therefore evident that the principle employed, which is rational in theory, is equally as sound in practice, and I do not believe the æsthetic results obtained could have been equalled by any other method of procedure.—*Items of Interest.*

